# Cognitive Dysfunction in Migraineurs

**DOI:** 10.3390/medicina58070870

**Published:** 2022-06-29

**Authors:** Tong Qin, Chunfu Chen

**Affiliations:** Department of Neurology, Shandong Provincial Hospital, Shandong University, Jinan 250021, China; quintessa7@163.com

**Keywords:** cognitive dysfunction, migraine, MoCA, psychological disorders, tension-type headache

## Abstract

*Background and Objectives:* Migraines are one of the most common types of primary headaches in neurology. Many studies to date have investigated cognitive impairment in migraineurs, but the results are inconsistent. This study aimed to investigate the cognitive function of migraineurs and explore the influencing factors. *Material and Methods:* A total of 117 patients with primary headaches (87 with migraine and 30 with tension-type headache (TTH)) and 30 healthy controls were enrolled. General information and data on headache clinical characteristics, and assessments of headache-related disability, psychological symptoms, and cognitive function were collected for statistical analysis. *Results:* The Montreal Cognitive Assessment (MoCA) total score and the scores of visuospatial and executive functions, language, and delayed recall in the migraine and TTH groups were significantly lower than those in the healthy control group (all *p* < 0.05). The MoCA total score did not correlate with Headache impact test-6, Migraine Disability Assessment Questionnaire, Patient Health Questionnaire-9, or Generalized Anxiety Disorder Questionnaire-7 scores in migraineurs (all *p* > 0.0125). The multiple linear regression analysis showed that age and duration of attack had a major influence on the overall and various fields of cognition in migraineurs. *Conclusion:* The study confirmed the impairment of cognitive function in patients with migraine and TTH, and found that the duration of attack had an effect on cognitive function in migraineurs.

## 1. Background

Migraines are one of the most common types of primary headaches in neurology. The results of the Global Burden of Disease survey showed that the impact of migraine on disability-adjusted life years of neurological disorders ranked second, only next to stroke, accounting for approximately 16.3% [1,2]. Migraine has a great influence on the work and life of migraineurs as a result of recurrent and severe pain. Migraineurs often complain of cognitive impairment. Psychological disorders such as anxiety and depression are common comorbidities of migraine. Cognitive dysfunction has become a focal problem in migraine. To date, many studies have investigated cognitive impairment in migraineurs, but the results are inconsistent. Most studies have shown impaired cognitive function in migraineurs [3,4,5], mainly in language, memory, visual space, executive function, and attention. However, a few studies did not find any cognitive differences between migraineurs and non-migraine controls, especially in longitudinal studies [6,7].

The discrepancy between the aforementioned findings might be explained by the following reasons: different clinical characteristics of the enrolled patients (age range, with aura or without aura, ictal or interictal phase, etc.), different sample sizes, different methods of neuropsychological assessment, and so forth. In addition, some studies did not control for the possible influence of other variables in the clinic, such as frequency of attacks, degree of pain, duration of each attack, treatment conditions, and so forth. Whether a link exists between cognitive dysfunction and clinical features in migraineurs is inconclusive. No current neuropsychological studies revealed that cognitive impairment in migraineurs was associated with the commonly used therapeutic drugs, although some migraine-preventive drugs (e.g., topiramate) might cause cognitive impairment [8,9]. Most studies found that psychological symptoms were not significantly associated with cognitive impairment in migraineurs [9,10,11,12]. However, a few studies have come to the opposite conclusion [3,13]. Since the conclusions of the studies are not the same, the relationship between cognitive dysfunction and psychological symptoms in patients with migraine still needs further research.

Tension-type headache (TTH) is one of the major types of primary headaches. Large differences exist in the pathogenesis and clinical characteristics between TTH and migraine. Patients with TTH are also affected by cognitive decline [14,15]. Current studies found that migraineurs had more pronounced cognitive impairment than patients with TTH [16,17,18]. Therefore, comparing migraineurs with patients with TTH and observing differences or associations between the two types of headache may be helpful.

The Montreal Cognitive Assessment (MoCA) is a widely used assessment tool for cognitive impairment. The MoCA is particularly suitable as a neuropsychological assessment tool for migraineurs due to its sensitivity to impairments in execution, attention, and visuospatial functions. Hence, the MoCA was used as the primary cognitive assessment tool in this study. This study aimed to investigate the cognitive impairment of migraineurs and explore the association between cognitive function and clinical features, headache-related disability, and psychological symptoms in migraineurs, providing a theoretical basis for a better understanding of migraine, preventing its progression, and providing tools for prognostic evaluation.

## 2. Materials and Methods

### 2.1. Participants

A total of 117 patients with primary headaches were enrolled, including 87 patients with migraine (70 without aura and 17 with aura) and 30 patients with TTH. The inclusion criteria were as follows: (1) diagnoses made for all study patients by referring to the International Classification of Headache Disorders 3rd edition (ICHD-3) [19], and patients who met the diagnostic criteria included in the migraine or TTH group; (2) aged 18–65 years; (3) medication status unchanged for the last month; and (4) signed the informed consent. The exclusion criteria were as follows: (1) diagnosed with other types of primary headaches or secondary headaches; (2) complicated with other types of primary headaches; (3) diagnosed with dementia or organic brain dysfunction (including neurological disorders affecting cognitive function, severe head trauma, etc.); (4) diagnosed with severe cardiovascular diseases, cerebrovascular diseases, or severe systemic diseases such as malignancy and hepatic and renal failure; (5) diagnosed with severe mental disorders such as schizophrenia and bipolar disorders; and/or (6) patients who refused enrollment and could not cooperate to complete the questionnaire.

Thirty patient attendants and physical examinees who had no history of headache, cardiovascular or cerebrovascular diseases, dementia, organic brain dysfunction, malignancy, hepatic failure, renal failure, mental disorders, or other major diseases were selected as the healthy control group.

### 2.2. Data Collection

The data were collected in the form of a questionnaire, scales, and so forth. The contents of the data that needed to be collected included general information, clinical features of headache, and assessments of headache-related disability, psychological symptoms, and cognitive functions. Headache-related disability was assessed using the Migraine Disability Assessment Questionnaire (MIDAS) and Headache Impact Test-6 (HIT-6). Psychological symptoms were assessed using the Patient Health Questionnaire-9 (PHQ-9) and Generalized Anxiety Disorder Questionnaire-7 (GAD-7). Cognitive functions were assessed using MoCA.

#### 2.2.1. Headache Characteristics

The headache characteristics mainly included the age of headache onset, course of headache, pain intensity, attack frequency, duration of each attack, with or without aura, accompanying symptoms, treatment, and so forth.

The pain intensity was assessed using the Numerical Rating Scale (NRS). A line segment was equally divided into 10 parts to evaluate the intensity of pain, with 0 being no pain, 1–3 mild pain, 4–6 moderate pain, 7–9 severe pain, and 10 being the worst. The participants scored the most common and severe pain of previous headache attacks based on subjective experience.

The attack frequency was evaluated by the average number of attacks per month in the last 3 months.

The duration of attacks was evaluated by the most common and the longest duration, and divided into 0.5–4 h, 4–12 h, 12–24 h, 24–72 h, and >72 h.

The preventive medications included β-blockers such as propranolol, calcium channel blockers such as flunarizine hydrochloride, antidepressants such as duloxetine, antiepileptics such as topiramate, Chinese patent drugs, and so forth.

#### 2.2.2. Assessment of Headache-Related Disability

MIDAS is a self-test questionnaire quantifying the degree of migraine-related disability over the 3-month period by evaluating the time loss due to headaches in three aspects: work and study, housework, and non-labor activities (family and social activities). The days of all losses were added to yield MIDAS scores with four-level severity: 0–5 as level I (minor or not frequent), 6–10 as level II (mild or frequent), 11–20 as level III (moderate), and ≥21 as level IV (severe).

HIT-6 is a questionnaire to assess the extent of headache impact on quality of life, mainly evaluating six aspects: pain, social function, role function, vitality, cognitive function, and psychological symptoms. The score range was 36–78 into four levels: ≤49 as level I (no or minor effect), 50–55 as level II (moderate effect), 56–59 as level III (significant influence), and ≥60 as level IV (severe influence).

#### 2.2.3. Assessment of Psychological Symptoms

PHQ-9 is a common clinical self-test scale of depression, including nine common depressive symptoms. The patients scored based on their own feelings in the last 2 weeks. Patients with scores 0–4 were classified as no depression, 5–9 as possible mild depression, 10–14 as possible moderate depression, 15–19 as possible moderate-to-major depression, and 20–27 as possible major depression.

GAD-7 is a common self-test scale of anxiety, including seven items of common anxiety symptoms. The patients scored according to the frequency of symptom occurrence in the last 2 weeks. Scores 0–4 indicated no generalized anxiety disorder, 5–9 mild generalized anxiety disorder, 10–14 moderate generalized anxiety disorder, and 15–21 severe generalized anxiety disorder.

#### 2.2.4. Assessment of Cognitive Function

MoCA is a widely used cognitive assessment scale in the clinic that assesses cognitive function in eight major domains: naming, memory, attention, language, abstraction, delayed recall, orientation, and visuospatial and executive functions, with a full score of 30. If the years of education are no more than 12, 1 should be added to the score, with final maximum scores of 30 and ≥26 considered normal.

### 2.3. Statistical Analysis

The collected clinical data were established into a database, and SPSS 25.0 software (IBM Corp. Released 2017. IBM SPSS Statistics for Windows, Version 25.0. Armonk, NY, USA: IBM Corp.) was used for statistical analysis.

The Shapiro–Wilk test was applied for normality testing. The enumeration data were described by *n* (%). The measurement data with a normal distribution were described by the mean ± standard deviation ($\bar{x}$ ± *s*). The measurement data with a skewed distribution were described by the range and median (*M*). When the distribution of each group was inconsistent, the range and median (*M*) were uniformly adopted.

When comparing three groups, one-way analysis of variance was used for measurement data with a normal distribution. The Kruskal–Wallis H test was used for measurement data with a skewed distribution and one-way ordinal enumeration data.

When comparing two groups, a *t* test or *t*’ test was used for measurement data with a normal distribution. The chi-square test or Fisher’s exact test was used for two-way disordered enumeration data. The Mann–Whitney *U* test was used for measurement data with a skewed distribution and one-way ordinal enumeration data.

The correlation analysis was performed by Spearman correlation analysis using a Bonferroni correction for multiple comparisons. The analyses of factors influencing cognitive function in migraineurs were performed using multiple linear regression. All hypothesis tests were two tailed, and *p* < 0.05 indicated a significant difference. The Bonferroni corrected significance level was 0.0125 (0.05/4) in analyzing the correlation between cognitive function and the degree of headache-related disability as well as the scores of psychological symptoms.

## 3. Results

### 3.1. General Information

The general conditions in the migraine, TTH, and healthy control groups are shown in Table 1. No significant differences were found in age, sex, or years of education among the three groups (all *p* > 0.05), suggesting that all three groups were comparable.

### 3.2. Headache Characteristics and Headache-Related Disability

The results of the comparison of headache characteristics and headache-related disability between patients with migraine and those with TTH are presented in Table 2. Both the most common and the severest NRSs in the migraine group were higher than those in the TTH group (*p* = 0.003 and 0.001, respectively). Both the most common and the longest attack durations in the migraine group were shorter than those in the TTH group (*p* = 0.025 and 0.044, respectively). The average number of headache days per month in the migraine group was less than that in the TTH group (*p* < 0.001). The HIT-6 score was higher in the migraine group than in the TTH group (*p* = 0.001).

### 3.3. Assessment of Cognitive Function

The results of the comparison of cognitive function scores among the migraine, TTH, and healthy control groups are presented in Table 3. Statistically significant differences were observed in the MoCA total score and the scores on the subtests of the visuospatial and executive functions, language, and delayed recall (all *p* < 0.05) among the three groups. The results of the pairwise comparisons among the three groups are shown in Table 4. The total MoCA scores in the migraine and TTH groups were significantly lower than those in the healthy control group (*p* = 0.001 and *p* < 0.001, respectively). The visuospatial and executive function scores in the migraine and TTH groups were significantly lower than those in the healthy control group (all *p* < 0.001). The language scores in the migraine and TTH groups were significantly lower than those in the healthy control group (*p* = 0.030 and 0.016, respectively). The delayed recall scores in the migraine and TTH groups were significantly lower than those in the healthy control group (*p* = 0.005 and 0.006, respectively).

### 3.4. Assessment of Psychological Symptoms

The results of the comparison of psychological symptom scores among the migraine, TTH, and healthy control groups are presented in Table 5. The differences in the PHQ-9 and GAD-7 scores among the three groups were statistically significant (all *p* < 0.001). Pairwise comparisons were performed among the three groups. The results are shown in Table 6. GAD-7 and PHQ-9 scores in the migraine and TTH groups were significantly higher than those in the healthy control group (all *p* < 0.001).

### 3.5. Correlations of Cognitive Function with Headache-Related Disability and Psychological Symptoms

The results of the correlation analyses between cognitive function and headache-related disability and the psychological symptoms in migraineurs are presented in Table 7. The correlations between the orientation score and the HIT-6, MIDAS, and PHQ-9 scores were statistically significant (*p* < 0.001, 0.003, and 0.003, respectively).

### 3.6. Factors Influencing Cognitive Function in Migraineurs

Multiple linear regression was used to analyze the influencing factors of the MoCA total and subset scores. The analysis results are shown in Table 8 and Table 9. The effect of age on the total MoCA score in migraineurs was statistically significant (*p* = 0.010). The total MoCA score decreased by 0.142 for every 1-year increase in age. The factors influencing the visuospatial and executive functions were age, aura, the most common attack duration, and the longest attack duration. The only factor influencing attention was age. The factors influencing language were age, the most common attack duration, and topiramate administration. The only factor influencing abstraction was the most common attack duration. The factors influencing delayed recall were age and the most common attack duration.

In multiple linear regression analysis of the factors influencing the MoCA total and subset scores in migraineurs, the most common attack duration and the longest attack duration were set as dummy variables using 4–12 h or 24–72 h as references, respectively. The analysis results are presented in Table 10.

## 4. Discussion

### 4.1. Headache Characteristics and Headache-Related Disability

In this study, no significant difference was found between the migraine and TTH groups in terms of age of onset or disease course. Since most patients with migraine and TTH had a good prognosis [20], the disease courses were mostly within 10–20 years.

We found that the patients in the migraine group had a higher intensity, a shorter duration of each attack, and less frequent attacks compared with those in the TTH group, which was in accordance with the clinical characteristics of migraine and TTH. Migraine is a moderate-to-severe headache, and headache attacks generally last for 4–72 h, whereas TTH is a mild-to-moderate headache, with an attack time of 30 min to seven days. The higher frequency of headache attacks in the TTH group might be due to the generally lower severity of TTH, and the patients who visited the clinic were mostly those with a higher frequency and a greater impact on their life. A population study [21] showed that patients with TTH less often sought medical help, and the consultation rate was 16% among patients with TTH compared with 56% among migraineurs. We refined headache severity and attack duration to the most common and most severe levels, which not only provided a more accurate and comprehensive assessment of a patient’s headache but also avoided patient neglect of usually less intense attacks and reduced patients’ recall bias due to the impressiveness of severe attacks.

HIT-6 scores were higher in the migraineurs than in patients with TTH; no significant difference was found in MIDAS scores between the two groups. The MIDAS is an assessment scale for migraine-related disability that quantifies the amount of time lost due to migraine over a 3-month period. It includes three dimensions: paid labor, housework, and family and social activity. The HIT-6 is a questionnaire mainly evaluating pain, social function, role function, vitality, cognitive ability, and psychological abnormalities. It was concluded that no significant difference existed between migraine and TTH in their effects on social function, but migraine had a greater subjective effect in other aspects such as pain, cognition, psychology, and vitality.

### 4.2. Cognitive Function

The results showed no significant difference in the overall level of cognitive function between patients with migraine and those with TTH. Both groups had lower levels of cognitive function compared with healthy controls, especially in visuospatial and executive functions, language, and delayed recall. However, no significant differences were observed in naming, attention, abstraction, and orientation between the patients and healthy controls.

The conclusion that migraineurs had cognitive impairment was consistent with previous findings [3,4,9], but various studies reached mixed conclusions with respect to the domains of cognitive decline. Many studies considered migraineurs to have a cognitive decline in memory and attention [3,9], which was supported by imaging- and electrophysiology-related studies as follows. Mickleborough et al. [22] found using fMRI (functional magnetic resonance imaging) that the activation of the key regions of the ventral anterior parietal attention network decreased in migraineurs during both intentional and reflective visuospatial orientation, indicating that migraineurs lacked inhibition of non-attentional events. Huang et al. [4] found that the latencies of the P3 components of Fz, Cz, and Pz in event-related potentials in migraineurs were prolonged. In addition, Wang et al. [22] found that the P3 amplitude of migraineurs without aura decreased, showing that migraineurs had frontal functional defects related to automatic attention switching. We did not find any decreased function in attention, which might be related to the small sample size, different evaluation technology and environment, and individual differences. In fact, some studies did not find cognitive impairment in migraineurs, especially in longitudinal studies [6,7].

The results showed that patients with TTH had cognitive impairment, which was consistent with previous findings [14,15]. Patients with TTH had dysfunction in logical reasoning and semantic processing, with a greater degree of mental impairment and easier distraction [15].

We found no significant difference in cognitive function between patients with migraine and those with TTH. Previous studies found that the perceptual organization ability of children with migraine was lower than that in children with TTH or healthy controls [16]. Also, their short-term visual memory and visuomotor integration ability were significantly lower than those of children with TTH [17]. Through a somatosensory time discrimination test, Vuralli et al. [18] found that the central sensory processing function of migraineurs was significantly damaged during headache attacks, while that of patients with TTH remained intact. We did not explore the differences in sensory perception between patients with migraine and those with TTH, which needs to be explored further.

### 4.3. Psychological Symptoms

We found no significant difference in the evaluation of psychological symptoms between patients with migraine and those with TTH. The scores of anxiety and depression in the two groups were higher than those in the healthy controls, which was consistent with the previous literature [15,23]. However, a study showed no significant difference in psychological symptoms between migraineurs without aura and healthy controls [9].

### 4.4. Correlations of Cognitive Function with Headache-Related Disability and Psychological Symptoms in Migraineurs

We found no correlation between the overall cognitive level of migraineurs and headache-related disability, which was different from the results reported in the previous literature. Gil-Gouveia et al. [10] found that cognitive symptoms related to migraine attacks were severe and disabling, and some of them were related to the subjective attack intensity and degree of disability. Cognitive performance should be used as a valuable secondary endpoint in acute migraine treatment trials. In this study, only the orientation score was related to headache-related disability.

Although orientation function was related to the depression score, the overall cognitive level of patients with migraine had nothing to do with the evaluation of psychological symptoms, which was consistent with the results of most previous studies [9,10,11,12]. However, a few studies found opposite results [3,13]. It was seen that the decline in orientation function was associated with social function and depression, but the decline in overall cognitive function had no serious impact on daily life and emotions in migraineurs.

### 4.5. Factors Influencing Cognitive Function in Migraineurs

We found that the overall cognitive level, visuospatial and executive functions, attention, language, and delayed recall of migraineurs were related to age. In addition, aura did not affect the overall cognitive function of migraineurs, but it was related to visuospatial and executive functions. The research results of Huang et al. [4] showed that the cognitive function of migraineurs with aura was significantly lower than that of migraineurs without aura, especially in visuospatial and executive functions, naming, memory, attention, and abstraction. We did not find any association of the cognitive function of migraineurs with the period of attack or the age of onset. However, Gil-Gouveia et al. [5] found that the cognitive function of migraineurs decreased during the attack period compared with that during the interval, especially in reading and processing speed, language memory, and learning, indicating reversible brain dysfunction, which might be related to the migraine or the result of acute pain processes in the brain. He et al. [24] found that the age of onset of migraine was independently related to the decline in cognitive function.

We did not find a correlation between cognitive function and headache intensity, but the attack duration could affect the overall cognitive level, visuospatial and executive functions, language, abstraction, and delayed recall in migraineurs. Studies found that the MoCA score of migraineurs was significantly correlated with the attack duration [4]. The results showed that the attack frequency did not affect the cognitive function of migraineurs, which was consistent with the results of Santangelo et al. [9]. However, the results of Huang et al. [4] showed that both MoCA executive function and calculation scores and a decline in the Rey–Osterrieth complex figure test recall score were associated with the frequency of migraine.

We found that topiramate administration had an effect on language function but not on the overall cognitive level of migraineurs. Topiramate is an antiepileptic drug, and its main adverse effects include impairment of memory and verbal learning ability. Cognitive impairment is less frequent in migraineurs treated with topiramate than in patients with epilepsy, which may be related to the small dose required to treat migraine, the younger age of migraineurs, and the difference in titration protocols [25] Ferreira et al. [8] found that cognitive impairment was present in patients with chronic migraine, independent of the comorbidities and medication.

In conclusion, age and attack duration had a wide range of effects on the overall cognitive level and various cognitive domains in patients with migraine. Aura mainly affected visuospatial and executive functions, and topiramate administration was mainly associated with language function impairment in patients with migraine.

### 4.6. Strengths and Weaknesses

The present study had the following advantages. First, when exploring the relationship between migraine and cognitive dysfunction, the main research subject of this study was the young and middle-aged population, which fit the epidemiological characteristics of the age of migraine onset and reduced the disturbance of age-related dementia to some extent. Second, patients with TTH were included in this study as a control group for migraine, and both types of headaches belonged to primary headaches with different pathogenesis. The TTH group, as one of the control groups, could exclude the confounding effect of long-term pain on cognitive function, thereby controlling the influencing factors of cognitive function of migraine on the pathogenesis of the disease itself. Third, a large number of variables in migraineurs were collected in this study, which allowed us to extensively explore the relationship between cognitive functions and other factors, such as clinical features, headache-related disability, and psychological disorders, and could effectively control the interfering effects of confounding factors when performing statistical analysis.

The present study had the following shortcomings. First, this study used questionnaire and scales to investigate the study subjects. The obtained information was based on the subjects’ anamnestic description of migraine history, which might be wrong due to recall bias and lead to an incorrect diagnosis. However, migraine is a chronic and disabling disorder with a long disease course and moderate-to-severe pain, which impressively affects patients’ social function and daily life. Therefore, recall bias is less likely. Second, the sample size of this study was small, and many variables were non-normally distributed. The nonparametric test was often used as a statistical method, which might lead to a decrease in the power of the test. Third, many longitudinal studies did not find cognitive impairment in patients with migraine, and further long-term follow-up was not performed in this study. Fourth, this study did not include supplementary examinations such as imaging, hemodynamics, and electrophysiology for the objective assessment of cognitive dysfunction in migraineurs. Fifth, the information on the use of antimigraine drugs was screened in this study by only selecting whether or not the patients received preventive treatment and whether or not they received topiramate. The related variables of acute-phase treatment were not selected, thus possibly underestimating the extent of the effect of antimigraine drugs and consequently influencing the accuracy of the results. However, most previous studies reported that cognitive decline in patients with migraine occurred during the natural course of migraine and was not significantly associated with the presence or absence of comorbidities and medication use.

## 5. Conclusions

Combined with the previous literature, the results of this study confirmed the impairment of cognitive function in patients with migraine. The study of cognitive dysfunction in migraineurs might help better understand the harm caused by migraine, thus providing evidence and direction for the early prevention of its progression and encouraging further prognostic research. The results of current studies on cognitive impairment in migraineurs are inconsistent, with few reports from prospective studies. Therefore, the association between migraine and cognitive dysfunction awaits further investigation.

## Figures and Tables

**Table 1:** General conditions in the migraine, TTH, and healthy control groups.

Indices	Migraine Group (*n* = 87)	TTH Group (*n* = 30)	Healthy Control Group (*n* = 30)	*p* Value
Age (year)	18–56 (34.0)	18–63 (36.0)	20–57 (41.5)	0.151
Female	57 (65.5)	15 (50.0)	15 (50.0)	0.170
Education (year)	0–17 (12.0)	3–16 (9.0)	7–16 (15.0)	0.061

Note: The enumeration data were described by *n* (%). The measurement data with a skewed distribution were described by range and median (*M*). TTH, tension-type headache.

**Table 2:** Headache characteristics and headache-related disability in the migraine and TTH groups.

Indices	Migraine Group (*n* = 87)	TTH Group (*n* = 30)	*p* Value
Aura	17 (19.5)	-	-
Attack phase	31 (35.6)	-	-
Age of onset (year)	6–54 (23.0)	6–55 (21.5)	0.743
Course of disease (year)	0.1–33 (10.0)	0.3–52 (6.5)	0.881
Most common NRS	2–9 (5.0)	2–6 (4.5)	0.003
Most serious NRS	3–10 (8.0)	2–10 (6.0)	0.001
Most common attack duration *	-	-	0.025
Longest attack duration *	-	-	0.044
Average number of headache days per month for the last 3 months (d)	1–30 (5.0)	1–30 (13.0)	<0.001
Taking topiramate	10 (11.5)	5 (16.7)	0.529
Receiving preventive treatment	28 (32.2)	10 (33.3)	0.908
HIT-6	64.5 ± 6.66	59.7 ± 7.77	0.001
MIDAS	0–170 (20.0)	0–270 (12.5)	0.375

Note: * The attack duration was divided into 0.5–4 h, 4–12 h, 12–24 h, 24–72 h, and >72 h. The enumeration data were described by *n* (%). The measurement data with a normal distribution were described by the mean ± standard deviation ($\bar{x}$ ± *s*). The measurement data with a skewed distribution were described by the range and median (*M*). HIT-6, Headache Impact Test-6; MIDAS, Migraine Disability Assessment Questionnaire; NRS, Numerical Rating Scale; TTH, tension-type headache.

**Table 3:** Scores of cognitive function in the migraine, TTH, and healthy control groups.

MoCA	Migraine Group (*n* = 87)	TTH Group (*n* = 30)	Healthy Control Group (*n* = 30)	*p* Value
Total score	7–30 (26.0)	16–30 (23.5)	24–30 (27.0)	<0.001
Visuospatial and executive functions	0–5 (4.0)	1–5 (4.0)	2–5 (5.0)	<0.001
Naming	0–3 (3.0)	1–3 (3.0)	1–3 (3.0)	0.569
Attention	0–6 (6.0)	2–6 (6.0)	4–6 (6.0)	0.298
Language	0–3 (2.0)	0–3 (2.0)	1–3 (3.0)	0.040
Abstraction	0–2 (1.0)	0–2 (1.0)	0–2 (1.0)	0.363
Delayed recall	0–5 (3.0)	0–5 (3.5)	1–5 (4.0)	0.007
Orientation	3–6 (6.0)	5–6 (6.0)	5–6 (6.0)	0.153

Note: The measurement data with a skewed distribution were described by the range and median (*M*). MoCA, Montreal Cognitive Assessment; TTH, tension-type headache.

**Table 4:** Pairwise comparisons of MoCA total scores and scores of visuospatial and executive functions, language, and delayed recall among the three groups (*p* values).

Group	MoCA Total Score		Visuospatial and Executive Functions		Language		Delayed Recall	
	Migraine Group	TTH Group	Migraine Group	TTH Group	Migraine Group	TTH Group	Migraine Group	TTH Group
Migraine group	-	-	-	-	-	-	-	-
TT group	0.120	-	0.151	-	0.490	-	0.554	-
Healthy control group	0.001	<0.001	<0.001	<0.001	0.030	0.016	0.005	0.006

Note: MoCA, Montreal Cognitive Assessment; TTH, tension-type headache.

**Table 5:** Scores of psychological symptoms in the migraine, TTH, and healthy control groups.

Indices	Migraine Group (*n* = 87)	TTH Group (*n* = 30)	Healthy Control Group (*n* = 30)	*p* Value
PHQ-9	0–25 (7.0)	0–22 (9.5)	0–13 (2.5)	<0.001
GAD-7	0–19 (5.0)	0–19 (6.0)	0–13 (1.0)	<0.001

Note: The measurement data with a skewed distribution were described by the range and median (*M*). GAD-7, Generalized Anxiety Disorder Questionnaire-7; PHQ-9, Patient Health Questionnaire-9; TTH, tension-type headache.

**Table 6:** Pairwise comparisons of PHQ-9 and GAD-7 scores among the three groups (*p* values).

Group	PHQ-9		GAD-7	
	Migraine Group	TTH Group	Migraine Group	TTH Group
Migraine group	-	-	-	-
TTH group	0.301	-	0.605	-
Healthy control group	<0.001	<0.001	<0.001	<0.001

Note: GAD-7, Generalized Anxiety Disorder Questionnaire-7; PHQ-9, Patient Health Questionnaire-9; TTH, tension-type headache.

**Table 7:** Correlations of cognitive function with the degree of headache-related disability and psychological symptoms (*r* value (*p* value)).

MoCA	HIT-6	MIDAS	PHQ-9	GAD-7
Total score	–0.150 (0.165)	–0.174 (0.107)	–0.052 (0.631)	–0.030 (0.782)
Visuospatial and executive functions	–0.130 (0.228)	–0.179 (0.096)	0.010 (0.929)	0.023 (0.836)
Naming	–0.183 (0.090)	0.121 (0.266)	0.024 (0.828)	–0.060 (0.580)
Attention	–0.096 (0.376)	–0.016 (0.881)	0.039 (0.717)	0.061 (0.577)
Language	–0.073 (0.502)	–0.067 (0.536)	0.041 (0.705)	0.035 (0.747)
Abstraction	–0.066 (0.541)	–0.077 (0.481)	–0.142 (0.188)	–0.164 (0.128)
Delayed recall	–0.092 (0.395)	–0.192 (0.075)	–0.033 (0.759)	0.015 (0.891)
Orientation	–0.368 (<0.001)	–0.314 (0.003)	–0.314 (0.003)	–0.228 (0.034)

Note: GAD-7, Generalized Anxiety Disorder Questionnaire-7; HIT-6, Headache Impact Test-6; MIDAS, Migraine Disability Assessment Questionnaire; MoCA, Montreal Cognitive Assessment; PHQ-9, Patient Health Questionnaire-9; *r* value, Spearman correlation coefficient with a Bonferroni corrected significance level of 0.0125 (0.05/4).

**Table 8:** Multiple linear regression analysis of the factors influencing the MoCA total score in migraineurs.

Indices	Unstandardized Coefficient *B*	Standard Error	*p* Value	95.0% Confidence Interval for the *B*
Age	–0.142	0.054	0.010	(–0.250 to –0.035)
Aura	–1.375	1.092	0.212	(–3.553 to 0.803)
Attack phase	0.087	0.900	0.923	(–1.707 to 1.882)
Age of onset	0.017	0.059	0.778	(–0.100 to 0.134)
Most common NRS	–0.414	0.355	0.247	(–1.122 to 0.294)
Most serious NRS	–0.113	0.314	0.719	(–0.739 to 0.512)
Most common attack duration *	-	-	-	-
Longest attack duration *	-	-	-	-
Average number of headache days per month for the last 3 months	–0.069	0.058	0.239	(–0.184 to 0.047)
Taking topiramate	–2.597	1.320	0.053	(–5.230 to 0.036)

Note: MoCA, Montreal Cognitive Assessment; NRS, Numerical Rating Scale. * Results are shown in Table 3, Table 4, Table 5, Table 6, Table 7, Table 8, Table 9 and Table 10.

**Table 9:** Multiple linear regression of the factors influencing the MoCA subsets in migraineurs.

MoCA Subsets	Age	Aura	Attack Phase	Age of Onset	Most Common NRS	Most Severe NRS	Most Common Attack Duration *	Longest Attack Duration *	Average Number of Headache Days per Month for the Last 3 Months	Taking Topiramate
Visuospatial and executive functions	–0.035, 0.013	–0.612, 0.030	0.015, 0.946	–0.013, 0.401	–0.075, 0.406	–0.007, 0.928	-	-	–0.016, 0.285	–0.443, 0.189
Naming	0.004, 0.664	0.053, 0.753	0.066, 0.629	–0.013, 0.138	0.023, 0.676	–0.054, 0.263	-	-	–0.002, 0.826	–0.083, 0.698
Attention	–0.020, 0.043	–0.328, 0.091	–0.025, 0.877	–0.004, 0.706	–0.025, 0.692	–0.062, 0.264	-	-	0.014, 0.178	–0.016, 0.946
Language	–0.034, 0.005	0.050, 0.832	0.283, 0.148	0.006, 0.608	–0.044, 0.566	0.004, 0.951	-	-	–0.018, 0.146	–0.758, 0.009
Abstraction	–0.011, 0.360	0.388, 0.114	0.162, 0.420	–0.001, 0.918	–0.053, 0.503	0.018, 0.796	-	-	–0.008, 0.526	–0.240, 0.416
Delayed recall	–0.059, 0.005	–0.369, 0.375	–0.011, 0.974	0.038, 0.094	–0.023, 0.866	–0.065, 0.583	-	-	–0.018, 0.421	–0.887, 0.080
Orientation	–0.003, 0.571	0.114, 0.290	–0.085, 0.307	0.004, 0.491	–0.049, 0.136	–0.046, 0.109	-	-	–0.001, 0.914	0.227, 0.064

Note: Multiple linear regression results are described as (*B*, *P*). MoCA, Montreal Cognitive Assessment; NRS, Numerical Rating Scale. * Results are shown in Table 10.

**Table 10:** Multiple linear regression analysis of the factors influencing MoCA total and subset scores in migraineurs: attack duration.

MoCA	Total Score		Visuospatial and Executive Functions		Naming		Attention		Language		Abstraction		Delayed Recall		Orientation	
Reference	4–12 h	24–72 h	4–12 h	24–72 h	4–12 h	24–72 h	4–12 h	24–72 h	4–12 h	24–72 h	4–12 h	24–72 h	4–12 h	24–72 h	4–12 h	24–72 h
Most common attack duration																
<4 h	–0.863, 0.472	2.610, 0.181	–0.529, 0.084	0.345, 0.482	0.282, 0.123	0.180, 0.547	0.017, 0.935	–0.286, 0.401	–0.082, 0.751	0.697, 0.097	0.195, 0.465	0.754, 0.083	–0.369, 0.416	0.582, 0.428	–0.078, 0.463	–0.007, 0.965
4–12 h	-	3.473, 0.059	-	0.874, 0.060	-	–0.102, 0.718	-	–0.303, 0.346	-	0.779, 0.049	-	0.559, 0.168	-	0.950, 0.169	-	0.070, 0.661
12–24 h	0.785, 0.568	4.257, 0.005	0.022, 0.949	0.896, 0.019	0.079, 0.703	–0.023, 0.923	0.248, 0.301	–0.055, 0.836	–0.037, 0.900	0.742, 0.023	0.272, 0.373	0.831, 0.014	0.507, 0.330	1.458, 0.011	–0.022, 0.854	0.048, 0.718
24–72 h	–3.473, 0.059	-	–0.874, 0.060	-	0.102, 0.718	-	0.303, 0.346	-	–0.779, 0.049	-	–0.559, 0.168	-	–0.950, 0.169	-	–0.070, 0.661	-
>72 h	–0.900, 0.694	2.573, 0.264	0.113, 0.845	0.987, 0.092	0.200, 0.567	0.098, 0.779	0.210, 0.616	–0.093, 0.823	–0.089, 0.856	0.690, 0.165	–0.181, 0.722	0.378, 0.459	0.404, 0.641	1.354, 0.122	0.163, 0.437	0.234, 0.261
Longest attack duration																
<4 h	6.844, 0.108	7.930, 0.074	2.411, 0.026	2.872, 0.011	0.219, 0.732	0.261, 0.695	0.528, 0.472	0.579, 0.448	–0.046, 0.960	–0.084, 0.929	–0.188, 0.841	–0.350, 0.719	2.462, 0.126	2.818, 0.092	0.003, 0.994	–0.188, 0.626
4–12 h	-	1.087, 0.469	-	0.460, 0.227	-	0.042, 0.854	-	0.051, 0.846	-	–0.038, 0.905	-	–0.163, 0.626	-	0.356, 0.531	-	–0.191, 0.152
12–24 h	1.442, 0.263	2.529, 0.070	0.378, 0.246	0.838, 0.018	0.335, 0.088	0.378, 0.075	0.349, 0.123	0.400, 0.099	–0.048, 0.862	–0.086, 0.771	0.367, 0.200	0.205, 0.505	0.146, 0.763	0.502, 0.338	0.031, 0.788	–0.160, 0.189
24–72 h	–1.087, 0.469	-	–0.460, 0.227	-	–0.042, 0.854	-	–0.051, 0.846	-	0.038, 0.905	-	0.163, 0.626	-	–0.356, 0.531	-	0.191, 0.152	-
>72 h	2.567, 0.133	3.654, 0.006	0.249, 0.562	0.709, 0.034	0.085, 0.742	0.127, 0.532	0.344, 0.247	0.395, 0.088	0.327, 0.371	0.288, 0.306	0.726, 0.057	0.564, 0.055	0.383, 0.551	0.739, 0.138	0.168, 0.261	–0.023, 0.845

Note: Multiple linear regression results were described as (*B*, *P*). MoCA, Montreal Cognitive Assessment.

## Data Availability

The data that support the findings of this study are openly available at 4 TU. ResearchData at https://doi.org/10.4121/17026823.v1 (accessed on 17 November 2021).
